# Current Trends and Limitations in Dengue Antiviral Research

**DOI:** 10.3390/tropicalmed6040180

**Published:** 2021-09-30

**Authors:** Juliet O. Obi, Hernando Gutiérrez-Barbosa, Joel V. Chua, Daniel J. Deredge

**Affiliations:** 1 Department of Pharmaceutical Sciences, University of Maryland School of Pharmacy, Baltimore, MD 21201, USA; juliet.obi@umaryland.edu; 2 Institute of Human Virology, University of Maryland School of Medicine, Baltimore, MD 21201, USA; Hgutierrezbarbosa@ihv.umaryland.edu

**Keywords:** antiviral targets, dengue virus, flavivirus, NS5, nucleoside inhibitors, non-nucleoside inhibitors, polymerase

## Abstract

Dengue is the most prevalent arthropod-borne viral disease worldwide and affects approximately 2.5 billion people living in over 100 countries. Increasing geographic expansion of *Aedes aegypti* mosquitoes (which transmit the virus) has made dengue a global health concern. There are currently no approved antivirals available to treat dengue, and the only approved vaccine used in some countries is limited to seropositive patients. Treatment of dengue, therefore, remains largely supportive to date; hence, research efforts are being intensified for the development of antivirals. The nonstructural proteins, 3 and 5 (NS3 and NS5), have been the major targets for dengue antiviral development due to their indispensable enzymatic and biological functions in the viral replication process. NS5 is the largest and most conserved nonstructural protein encoded by flaviviruses. Its multifunctionality makes it an attractive target for antiviral development, but research efforts have, this far, not resulted in the successful development of an antiviral targeting NS5. Increase in structural insights into the dengue NS5 protein will accelerate drug discovery efforts focused on NS5 as an antiviral target. In this review, we will give an overview of the current state of therapeutic development, with a focus on NS5 as a therapeutic target against dengue.

## 1. Introduction

Dengue is an arthropod-borne disease caused by the dengue virus (DENV), which is primarily transmitted by *Aedes aegypti* mosquitoes [1]. Dengue fever is the most prevalent mosquito-borne disease worldwide, with the virus circulating mainly in tropical and subtropical regions including Southeast Asia, the Americas, Africa, Western Pacific, and Eastern Mediterranean regions [2]. In 2013, dengue incidence was estimated to be approximately 400 million dengue infections annually, with 96 million cases manifesting clinically [3]. Dengue is endemic in over 100 countries and affects about 2.5 billion people living in the tropics and subtropics [4]. Although the mortality rate of severe dengue is relatively low–approximately 22,000 deaths annually from dengue shock syndrome (DSS) [5]–the disease has become a global health concern in the last few decades due to increasing geographic expansion of *Aedes aegypti* mosquitoes [6]. This expansion stems from global warming trends which increase the potential for dengue epidemics in temperate regions, increased urbanization, global trade, increased human travel, and the lack of effective vector control in dengue endemic regions [7,8,9].

Currently, there are no antivirals developed to treat dengue infection, and treatment remains supportive [10]. Although a DENV vaccine has recently been used in some countries, its indication is limited due to risk of severe dengue in certain populations [11]. This has led to calls for intensified research efforts for the development of a novel vaccine, therapeutics, and vector-control strategies against dengue, for better prevention and control. This review summarizes current research efforts in dengue antiviral research and development, with a focus on NS5 as a therapeutic target against dengue. For that purpose, we will first provide an overview of disease burden, epidemiology, clinical manifestations, and give an introductory overview of the current state of therapeutic efforts against the dengue virus. In a second section, we will explore the biology of dengue virus including the genome structure and viral life cycle. Finally, we will explore, in greater detail, the current efforts to develop antivirals against dengue with focus on NS5.

### 1.1. Disease Burden, Clinical Importance, and Manifestations

Although current research efforts in the development of effective vaccines and therapeutics against dengue are promising, the global burden of the disease is yet to be fully elucidated. The increase in geographical distribution of the virus is a major concern and adversely impacts both global health and economy in the future [8]. Many tropical and subtropical regions currently bear the disease and economic burden of dengue, with Asia accounting for 70% of the global disease burden, followed by Latin America and Africa [3]. The disease burden in Africa is known to be largely underestimated due to poor records of dengue occurrence data and from dengue being masked by other flu-like diseases or malaria, which have similar symptoms [12,13]. Nevertheless, a consistent increase in dengue epidemics has been observed in Africa and the Pacific and Indian Oceans in the last four decades [14,15].

The international economic burden of dengue is still loosely estimated. However, the assessment of disability-adjusted life years, provides evidence for the dengue economic burden, comparable to that of hepatitis B virus infections and upper respiratory tract infections [16]. The worldwide annual cost of dengue was estimated to be approximately USD 9 billion [17], with an average of USD 2.2 billion in the Americas (between 2000 and 2007), USD 1.2 billion in South-East Asia (between 2000 and 2010), and USD 76 million in Africa [17,18]. These estimates are predicted to increase in future years [19], hence the need for constant public health surveillance efforts in the estimation of the global burden and geographic expansion of dengue.

Dengue typically presents as an acute febrile illness often accompanied by headaches, retro-orbital pain, arthralgia, myalgia, and in some cases, with transient morbilliform rashes and petechiae. The most common laboratory abnormalities include leukopenia, thrombocytopenia, and liver enzyme elevation. Dengue is a self-limiting disease with an incubation period of around four to seven days, with viremia usually occurring during a febrile episode. Although majority of dengue cases are either asymptomatic or mild, approximately 500,000 people infected per year develop potentially life-threatening severe diseases, such as dengue hemorrhagic fever (DHF) and Dengue Shock Syndrome (DSS) [3]. Severe dengue is characterized by an abrupt onset of hemorrhagic manifestations with or without shock during periods of defervescence. In hyperendemic regions of the world, where all four DENV serotypes are circulating, the incidence of DHF/DSS is about 10- to 100-fold higher—particularly for those who develop secondary DENV infections. Although mortality rates can be mitigated with early supportive measures, dengue outbreaks continue to be a major public health problem that has a significant socioeconomic and healthcare impact in affected populations [20].

### 1.2. Viral Serotypes and Antibody-Dependent Enhancement (ADE)

Dengue virus has four different serotypes (DENV 1–4) based on structural antigens which induce type-specific antibodies upon infection [6]. A fifth serotype (DENV5), which is currently limited to only an outbreak in Sarawak, Malaysia, has also been identified [21,22]; however, its genome sequence is yet to be reported. Infection with any serotype often results in long-term immunity against the same serotype (homotypic immunity). Immunity against the other serotypes (heterotypic immunity) occur for a short period of time only [23], with cross-protection lasting for about six months [24]. Exposure to one DENV serotype is known to increase the risk of severe dengue infection following secondary infection with another DENV serotype, a phenomenon attributed to antibody-dependent enhancement (ADE) [25]. This ADE phenomenon can explain the increased disease severity observed during secondary infections with another serotype whereby non-neutralizing crossreactive antibodies bind to heterologous DENV. This binding facilitates viral entry through Fc receptors expressed on target cells, including dendritic cells, monocytes, and macrophages [26,27], and resulting in increased viral uptake and subsequent replication within the target cells [28,29]. Consequently, ADE results in higher viral load, and causes vascular leakage induced by a combination of pro-inflammatory and anti-inflammatory responses [30], ultimately leading to severe dengue shock syndrome [31].

All four major DENV serotypes principally causes similar clinical symptoms. However, certain biological differences occur among them [32,33]. These include, but are not limited to, epidemic potential, transmission efficiency, disease severity, host immunity associations between specific serotypes or genotypes, and conditions that favor the displacement of one genotype by another [34,35]. A 30% divergence occurs in the DENV polyprotein among the four serotypes, with many genotypes within a serotype identified in different geographic locations [34]. This led to the hypothesis that some DENV serotypes have greater epidemic potential and virulence compared to others [36,37]. The differences in epidemic potential have been mostly ascribed to genetic changes which result in amino acid changes in the nonstructural (NS) proteins [8].

### 1.3. Dengue Vaccines and Antiviral Agents: Current State

To date, no antiviral agent or universal vaccine is available to treat or prevent dengue. The only dengue vaccine available on the market is CYD-TDV, which was developed by Sanofi Pasteur (marketed as Dengvaxia^®^) [38] and is currently approved in 20 countries in Latin America, Asia, and Australia [21]. CYD-TDV is a live-attenuated vaccine, effective for the prevention of severe infection in previously infected people. However, this vaccine increases the risk of severe dengue in individuals who have not been previously infected (dengue-naïve individuals) [22]. This limits its use to seropositive individuals only and stresses the need for a universal vaccine to prevent infection in seronegative individuals, especially children [10]. There are several dengue vaccine candidates currently in clinical development, including live-attenuated tetravalent chimeric vaccines (Takeda’s TDV and United States [U.S.] National Institute of Health/Butantan’s TV003/TV005 and LAV Delta 30), a recombinant vaccine (Hawaii Biotech Inc./Merck’s DEN-80E), and a DNA vaccine (D1ME100 being developed by the U.S. Naval Research Center) [39,40,41].

Several antiviral candidates have been developed against dengue, but none have been successful to effectively treat dengue infection [42,43]. There is an urgent need for the development of therapeutics against dengue, as treatment is still primarily based on supportive therapy, including the use of analgesics and fluid replacement [3]. Other approaches, including artificial intelligence (AI) and computational analyses are currently being developed with great potential for advancing and accelerating dengue immunotherapeutic discovery [44]. Additionally, recent advances in molecular and structural virology are increasingly shedding more light on NS proteins, particularly the NS5 protein [45]. This makes flaviviral NS5 a very promising target for antiviral development.

## 2. Dengue Viral Life Cycle and Proteins

### 2.1. Genome Structure and Organization

**Figure 1:**
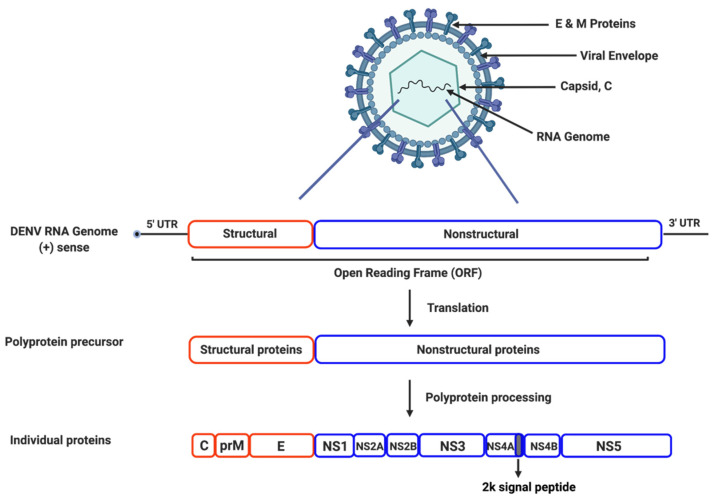
Genome Structure and Organization of the Dengue Virus. The dengue virus is spherical in shape, enveloped, and contains a positive-sense, single-stranded RNA genome. The genome encodes a precursor polyprotein, with an open-reading frame ~11 kb in length. Following viral entry into the host cell, the polyprotein is cleaved into three structural proteins (capsid, pre-membrane, and envelope), and seven nonstructural proteins (NS1, NS2A, NS2B, NS3, NS4A with a 2k signal peptide at the C-terminal, NS4B, and NS5).

Dengue viruses are enveloped, single-stranded, positive-sense RNA viruses of the Flaviviridae family. All four DENV serotypes are spherical, enveloped viral particles, approximately 500 Å in diameter [46]. Their genome consists of approximately 11,000 nucleotides and encodes a precursor polyprotein, which undergoes proteolytic processing to generate three structural proteins (capsid protein C, precursor membrane protein prM, and an envelope protein E) and seven nonstructural (NS) proteins including NS1, NS2A, NS2B, NS3, NS4A, NS4B, and NS5 (Figure 1) [5]. The structural proteins are part of the mature viral particle and are not involved in viral genome replication. The NS proteins are expressed only in dengue infected cells and are mainly responsible for viral replication [47,48,49].

The genome open reading frame (ORF) is flanked by two untranslated regions (UTRs): a 5′-UTR of approximately 95–135 nucleotides, which contains a type I cap like cellular mRNA; and a 3′-UTR of approximately 114–650 nucleotides, which lacks a poly(A) tail but ends in a conserved stem-loop (SL) secondary structure. Both UTRs are required for efficient viral translation and replication [50,51]. The 5′-UTR contains a large stem loop A (SLA) of about 70 nucleotides, which has been shown to promote viral RNA synthesis through interactions with NS5 [52]. Additionally, both UTRs contain complementary Upstream AUG Regions (known as UAR) and cyclization sequences (CS) that hybridize for genome cyclization and RNA synthesis to occur [50].

### 2.2. Viral Life Cycle

**Figure 2:**
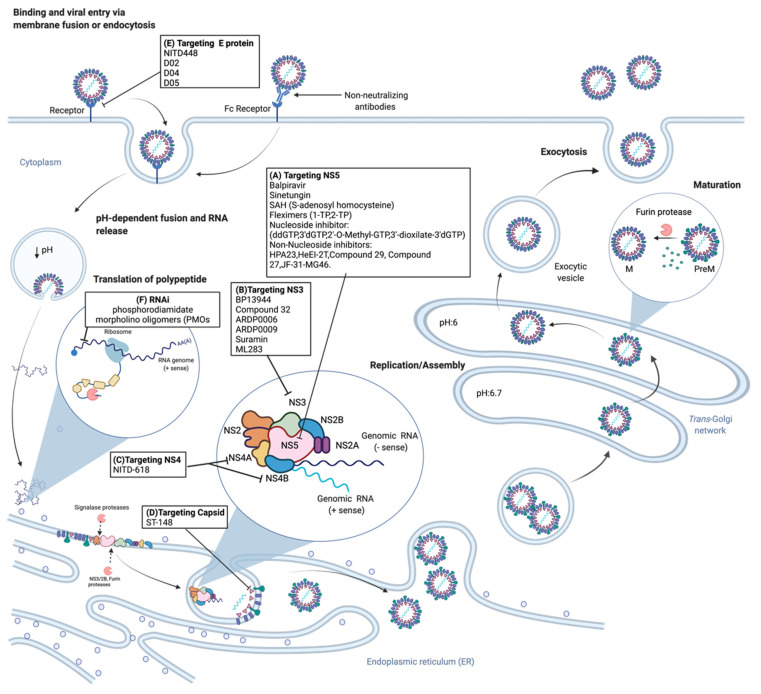
Life Cycle of the Dengue Virus and Antiviral Targets. The viral membrane attaches to host cell receptors (DC-SIGN, CLEC5A, heparan sulfate receptors) and viral entry occurs through receptor-mediated endocytosis. The viral genome is released into the cytoplasm followed by translation of the polyprotein encoding the open reading frame. Polyprotein processing leads to its cleavage into structural and nonstructural (NS) proteins by host proteases and the NS2B-NS3 viral protease. Viral replication occurs on the endoplasmic reticulum by NS proteins followed by viral assembly and trafficking of immature viral particles to the trans-Golgi network (TGN). The acidic environment of the TGN facilitates viral maturation and subsequent release of mature viral particles from the host cell. A list of inhibitors against targets at different stages of the viral cycle are indicated. DC-SIGN, dendritic cell-specific intracellular adhesion molecule-3-grabbing non-integrin; CLEC5A, human C-type lectin-like molecule.

The dengue virus life cycle consists of multiple steps including viral entry, viral replication, viral assembly, and viral release (Figure 2). The flavivirus life cycle is initiated by the fusion of the viral membrane with the host plasma membrane. This is followed by endocytosis of the virus into an endosome. The low pH of the endosome triggers a viral glycoprotein-mediated fusion of viral and host cellular membranes, permitting disassembly of the virion and subsequent release of the viral RNA genome into the cytoplasm [53]. The cell surface receptor, through which receptor-mediated endocytosis occurs during DENV viral entry, is yet to be fully identified. However, proposed host cellular receptors include dendritic cell-specific intracellular adhesion molecule-3-grabbing non-integrin (DC-SIGN), glycoproteins like heparan sulfate receptors, or mannose receptors [54,55,56]. Human C-type lectin-like molecule (CLEC5A) has also been proposed to function as a crucial macrophage receptor for DENV. Mice studies have also shown that it functions as a proinflammatory receptor for DENV [57,58].

After the viral genome is released into the cytoplasm, the positive-strand viral RNA is immediately translated into a single polyprotein. The translated polyprotein is then cleaved by viral and cellular proteases into three structural, and seven nonstructural proteins. Synthesis of a negative-strand intermediate then proceeds, which acts as a template for the synthesis of new positive-strand viral RNAs. The nonstructural proteins replicate the genomic RNA by going through several rounds of transcription to produce many viral genomes and subsequently viral proteins [59].

Viral assembly occurs at the endoplasmic reticulum (ER), where the capsid proteins and newly made viral RNA are enveloped by the ER membrane and glycoproteins to produce immature viral particles. Immature viral particles move through the secretory pathway and the Trans-Golgi Network (TGN), which has an acidic environment. The acidic environment of the TGN causes the precursor membrane protein (prM) to be processed by a furin host protease into the mature protein (M) [60]. The viral maturation process is known to be indispensable for infectivity, and studies suggest that prM shields the envelope proteins from premature fusion and pH-induced reorganization during viral secretion [61,62,63]. Following successful maturation of the virus, viruses are then released from the cell [5].

## 3. Dengue Antiviral Research and Development

### 3.1. Classical Antiviral Targets

Research efforts into the development of antiviral agents against dengue have intensified in the last few years since there are still no available treatments to date. Classical targets for the development of small molecule inhibitors include NS3 protease, NS3 helicase, NS4B, and NS5 proteins [64]. NS3 and NS5 proteins are thought to be the most crucial targets for antiviral development because of their indispensable enzymatic activities in the viral replication process. NS3 has multiple enzymatic activities including serine protease, nucleoside triphosphatase (NTPase), 5′-RNA triphosphatase, and helicase activities [65,66], while NS5 has methyltransferase (MTase) and RNA-dependent RNA polymerase (RdRp) activities [67]. Table 1 shows a list of compounds that target the dengue virus, for which crystal structures with the target was solved, divulging a binding site, and potentially supporting a mechanism of action.

#### 3.1.1. NS3 Protease

NS3 is the second largest flaviviral protein after NS5 (approximately 69 kDa), and it plays an important role in the viral replication cycle. It consists of two domains: an N-terminal protease domain responsible for cleaving the viral polyprotein precursor into individual proteins; and a C-terminal RNA helicase domain involved in DENV genome replication and viral RNA synthesis. The NS3 protease requires NS2B as a cofactor, to function properly. Studies have also shown that the NS3 protease is catalytically inactive in vitro, corroborating its need to bind to NS2B for proper folding and enzymatic activity [65,66].

Several inhibitors have been examined for use as NS2B-NS3 protease inhibitors (Table 1). Compound BP13944 for example, was shown to be a dengue protease inhibitor, through high-throughput screening (HTS) of over 50,000 compounds [68]. The compound inhibited dengue viral replication in all DENV serotypes with no apparent toxicity. Compound **32**, a keto amide was examined and shown to inhibit dengue viral replication in a dose-dependent manner [69]. Two anthracene-based compounds, ARDP0006 and ARDP0009, were identified through virtual screening and inhibited DENV-2 replication in cell culture studies [70]. Aprotinin was also identified, and studies showed that it binds to the NS3 protease pocket with high specificity and prevents the substrate from accessing the NS3 protease active site [71]. With many compounds identified as NS3 protease inhibitors, only few of them are effective for potential drug development against dengue, mostly because of their weak binding to the NS3 protease [72].

#### 3.1.2. NS3 Helicase

The C-terminal RNA helicase domain of NS3 is known to participate in the genome replication and RNA synthesis process with other NS proteins like NS5. The NS3 helicase activity is important for the fusion of secondary structures at the untranslated regions prior to initiation RNA synthesis. It is also responsible for unwinding dsRNA intermediate products formed during viral RNA synthesis, prior to the capping of the positive-strand RNA [66].

Many compounds identified as inhibitors against DENV NS3 are focused on the NS3 protease domain. This is more due to the crystal structures of the DENV NS3 helicase domain lacking binding pockets for potential small molecule inhibition [72,90]. Nonetheless, some compounds have been reported to have activity against DENV by inhibiting the NS3 helicase. For example, suramin, a polysulfonated compound with anthelminthic activity was shown to inhibit DENV NS3 helicase in a non-competitive manner [91,92]. In addition, analogues of the ML283 compound (which inhibits hepatitis C virus NS3 helicase) were identified as inhibitors of DENV NS3 helicase [91].

#### 3.1.3. NS4B

NS4B is a 27 kDa integral membrane protein with high hydrophobicity. It is fairly conserved among flaviviruses (approximately 40% sequence homology) [93] and is known to play a role in preventing host immune response following viral infection. While the NS4A protein triggers membrane reorganization and autophagy to improve the viral replication process, NS4B suppresses interferon α/β signaling and NS3 helicase activity resulting in regulation of the host’s immune cell response [65].

The NS4 proteins (NS4A and NS4B) do not have any reported enzymatic activity during viral RNA replication. However, they are known to be important in flavivirus replication and host interactions. NITD-618, a compound selected from a library of over 1.5 million molecules was found to be active against NS4B for all four DENV serotypes [94]. A new inhibitor against DENV NS4B, SDM25N was also identified, where it inhibits NS4B by restricting genomic RNA replication [95].

#### 3.1.4. NS5

**Figure 3:**
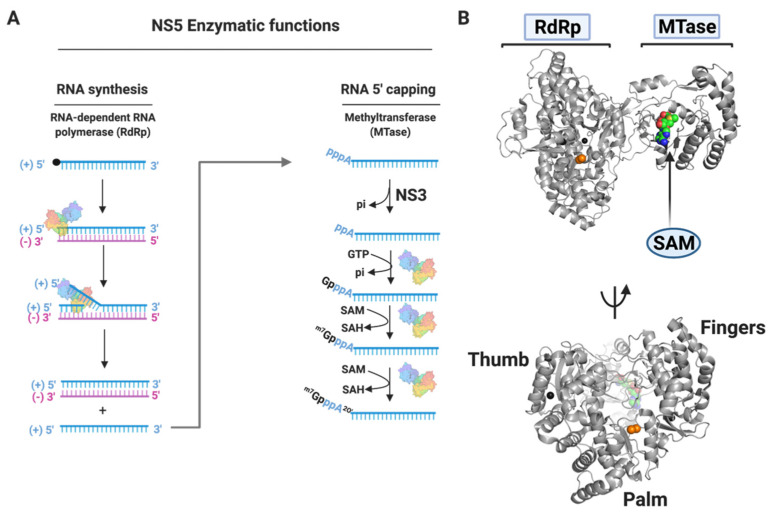
The Enzymatic Functions of Dengue NS5. NS5 plays a major role in the flaviviral replication complex including RNA synthesis, RNA capping, and RNA methylation. The exact sequence of events is not yet fully understood, but a general scheme (**A**) is widely accepted. The (+) strand genomic RNA is used by the NS5 RdRp as a template to synthesize a (−) strand RNA which results in a replicative dsRNA intermediate. The (−) strand of the dsRNA intermediate then serves as a template for (+) strand synthesis, after which the dsRNA product is released and used for additional synthesis of (+) strands. Newly synthesized (+) strands are capped and methylated to form (+) strands of genomic RNA. The 5′-RNA capping begins with the triphosphatase activity of the NS3 helicase, where the terminal phosphate at the 5′-end of the (+) strand RNA is removed leading to a diphosphorylated RNA. The NS5 methyltransferase (MTase) caps the RNA through its guanylyl-transferase activity, where guanine monophosphate (GMP) is transferred to the 5′-end of the diphosphorylated (+) strand RNA from GTP. The capped RNA is methylated by the NS5 MTase via two sequential methylations: an initial methylation at the N7 position of the guanine cap, and a subsequent methylation at the 2′O position of the first RNA nucleotide. S-adenosyl-L-methionine (SAM) is utilized as a methyl donor in both methylations and is converted to S-adenosyl-L-homocysteine (SAH) as a by-product of the reactions. A cartoon representation (**B**) of a crystal structure of the full-length dengue serotype 2 NS5 is shown with the canonical features of the NS5 RdRp. PDB ID: 5ZQK.

NS5 is the largest (approximately 100 kDa) and most conserved nonstructural protein encoded by flaviviruses, with over 75% sequence homology across all four DENV serotypes [67,71]. It is a key component of the viral replication complex, with multiple enzymatic and biological functions. NS5 contains an N-terminal methyltransferase (MTase) domain, responsible for synthesis of the 5′ RNA cap and methylation, and a C-terminal RNA-dependent RNA polymerase (RdRp) domain, responsible for viral RNA synthesis [96,97] (Figure 3). The C-terminal RdRp domain initiates RNA synthesis through a *de novo* mechanism, different from a primer-dependent mechanism employed by other viral polymerases. Flaviviral RdRp has a canonical right-hand structure, like other polymerases with palm, fingers, and thumb subdomains [67,98,99]. The palm subdomain has the most conserved structure among all known polymerases, and it contains the active site for polymerization. Flaviviral RdRps have two anti-parallel β-strands surrounded by eight α-helices, and three functional motifs which play a role in RNA synthesis. The fingers subdomain consists of a core and two fingertips, one of which connects to the thumb subdomain. The thumb subdomain also consists of eight α-helices and two anti-parallel β-strands with an interface that constitutes the motif E. Most importantly, the thumb subdomain consists of a loop that protrudes from the thumb towards the polymerase active site [98]. This priming loop is a unique feature of polymerases that carry out *de novo* RNA synthesis [100].

NS5 plays a fundamental role in the flaviviral replication cycle, making it a very important target for the development of antivirals against dengue. Apart from performing viral RNA synthesis, NS5 interacts with other viral proteins like NS3 and host proteins to carry out its replication functions [101]. It interacts with stem loop A (SLA) at the 5′ end of the dengue viral RNA to promote viral RNA synthesis [102]. It also carries out biological functions by binding to, and promoting the degradation of hSTAT2, to suppress type I interferon response [103]. Many antiviral agents have been evaluated against NS5 activity by either inhibiting its MTase or RdRp activity at different viral replication stages.

Sinefungin, a SAM (S-adenosyl-L-methionine) analogue was reported as a broad-spectrum DENV inhibitor. It binds to the SAM site in the DENV MTase domain in a similar fashion as SAM but does not undergo the same interactions with the protein as SAM does. However, MTase interaction assays showed Sinefungin’s affinity to be six times greater than that of SAM. Other DENV NS5 inhibitors that function as MTase inhibitors include SAH (S-adenosyl homocysteine), compound **10**, and GMP (guanosine monophosphate). These inhibitors, together with Sinefungin failed to show good progress because of their cell non-permeability [104]. Ribavirin, a synthetic guanosine analogue shown to inhibit HCV replication, displayed activity against DENV MTase but was later shown to be ineffective as a DENV prophylactic drug (Table 1) [105,106].

A novel group of flexible nucleoside analogs known as “fleximers” were recently developed and were shown to have antiviral activity against filoviruses such as the Ebola virus [107], coronaviruses (such as severe acute respiratory syndrome coronavirus (SARS-CoV) [108]), and flaviviruses (including Zika virus and Dengue virus). These fleximers have a “split” purine nucleobase which contributes significantly to the nucleoside scaffold activity [109,110,111]. Several fleximers of the FDA-approved acyclic nucleoside acyclovir, which is used to treat herpes infections have been developed. Recent studies have shown that some of these fleximers that have been developed (1-TP and 2-TP) inhibit the MTase activity of ZIKV and DENV, with very weak activity against the DENV RdRp even at high concentrations [79]. Fleximers have therefore shown to be promising nucleoside analogs which can target dengue NS5, with a low possibility of viral resistance against them [112].

Flaviviral RdRps are proposed to be the most important drug targets, because of their indispensable function in viral replication and most importantly because human host cells lack the viral RdRp [98]. DENV RdRp replicates the viral RNA genome in the absence of a primer strand (*de novo* replication mechanism). A complementary (−) RNA strand is first synthesized from the (+) RNA template strand to form a dsRNA duplex [113,114]. The dsRNA duplex is subsequently used as a template for the synthesis of additional (+) RNA strands which can either be used as mRNA for protein translation or packaged into new virions [84,97,101].

Small molecule inhibitors against viral RNA polymerases are generally divided into nucleoside inhibitors and non-nucleoside inhibitors based on their mechanism of inhibition.

#### Nucleoside Inhibitors

Nucleoside analog inhibitors (NI) are chemically modified analogs of endogenous nucleosides which can either block viral replication by disrupting DNA and RNA synthesis, or inhibit viral enzymes involved in nucleoside/nucleotide metabolism [115]. They are commonly used small molecule inhibitors for the treatment of viral infections including human immunodeficiency virus (HIV), hepatitis B virus (HBV), herpes viruses, and HCV [112]. Some of the approved nucleoside analogs used to treat these infections include tenofovir (HIV) [116,117], sofosbuvir (HCV) [118], and lamivudine/entecavir (HBV) [119,120]. Many potent inhibitors are currently being developed for HCV treatment including nucleoside analogs. Since arthropod-borne flaviviruses are closely related to HCV, nucleoside analogs, especially those developed against HCV, are promising targets that can be repurposed for the treatment of flaviviral infections, including dengue [121].

Nucleoside inhibitors, which target flaviviral RdRps, are very attractive for drug development because humans lack the RdRp enzyme leading to less off-target effects [115,121,122,123]. They target the polymerase active site which is situated in the palm subdomain of the RdRp [124]. The mechanism of action of nucleoside analog inhibitors involves the premature termination of viral nucleic acid synthesis [125]. They are generally converted into nucleosides following intracellular phosphorylation by host cell kinases [98]. This is followed by the incorporation of their 5′-triphosphate metabolites into the viral RNA nascent chain which occurs in a competitive fashion. The incorporation leads to the formation of nonfunctional viral RNA chains because of the premature termination of the elongating nascent viral RNA [126]. Nucleoside analog inhibitors, which target flaviviral RdRps functions as “nonobligate chain terminators”, display a 3′-hydroxyl group which is conformationally hindered reducing their ability to form a phosphodiester linkage with incoming nucleoside triphosphates [125]. Nucleoside inhibitors which function as obligate chain terminators lack the 3′-hydroxyl group. Nucleoside reverse transcriptase inhibitors used for the treatment of HIV infections are examples of obligate chain terminators [127].

Few compounds have been reported to date as nucleoside analog inhibitors against flaviviral RdRps. GTP analogs including ddGTP, 3′dGTP, 2′-O-methyl-GTP, and 3′-dioxolane 3′dGTP have been shown to weakly inhibit DENV2 RdRp activity through in vitro assays [97]. Further development of novel nucleoside analogs effective against flaviviruses like dengue remains in need.

#### Non-Nucleoside Inhibitors

Non-nucleoside analog inhibitors (NNI) do not bind to the putative active site of flaviviral RdRps. Instead, they bind to allosteric pockets or surface cavities of their target polymerase. They can induce a conformational change when they bind allosterically, resulting in an inactive polymerase. NNIs can also trap their target polymerase in a functional conformation but block an essential conformational transition from initiation to elongation required during RNA synthesis [98]. Allosteric inhibition using non-nucleoside inhibitors have shown to be an effective strategy for the inhibition of HCV RdRp activity and HIV reverse transcriptase activity [128]. However, only a few allosteric inhibitors have been described for DENV RdRp.

One of the first NNIs reported to inhibit DENV-2 RdRp activity is ammonium-21-tungsto-9-antimoniate, also known as HPA23 [129]. It was also previously reported to inhibit the reverse transcriptase activity of HIV by competing with the nucleic acid template [130], which does not make it a true allosteric inhibitor. A class of pyridobenzothiazole-based compounds including HeEI-2Tyr was reported to inhibit DENV RdRp activity and antiviral activity in DENV cell culture in the low micromolar range [81]. Their binding mechanism was shown to be similar to NITD-107 which binds to the RNA template tunnel of the polymerase (between the fingers subdomain and the priming loop), and locks the RdRp in a closed conformation, inhibiting DENV viral RNA synthesis [84].

A novel allosteric pocket of DENV RdRp (termed “N pocket”) was recently identified at the interface of the thumb and palm subdomains, close to the RdRp active site. Since the characterization of this allosteric binding site, potent compounds, which inhibited DENV 1-4 replication in many cell-based assays, including compound **29**, compound **27**, and JF-31-MG46 (Table 1) have been designed [80,82,85]. DENV allosteric N-pocket inhibitors are quite like the HCV polymerase site III (palm I) non-nucleoside inhibitors [131]. However, their binding affinities are weaker compared to the HCV class III inhibitors. The DENV N-pocket lacks the HCV primer grip wall, which stabilizes HCV inhibitor binding. Furthermore, the C-terminal loop of the HCV RdRp penetrates the active site and is involved in RdRp activity, unlike that of DENV, which is disordered in many crystal structures reported. The absence of these regions in DENV have been suggested to be the reason for the weaker binding affinities of DENV for the N pocket inhibitors compared to HCV site III inhibitors which form additional contacts with the primer grip wall and the C-terminal loop [132].

**Figure 4:**
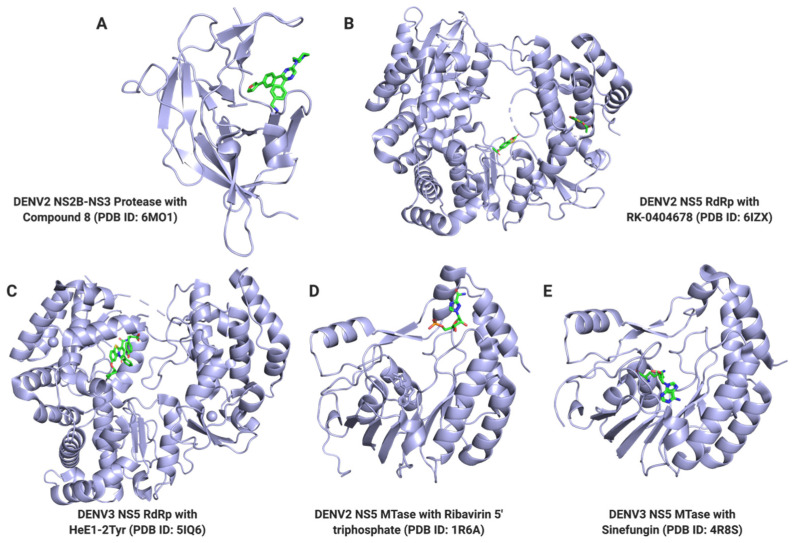
Representative Crystal Structures of NS3 and NS5 with Small Molecule Inhibitors. Cartoon representations of the (**A**) NS2B-NS3 protease, (**B**,**C**) NS5 RNA-dependent RNA polymerase (RdRp), and (**D**,**E**) NS5 methyltransferase (MTase) crystal structures are shown with inhibitor-bound small molecule (green sticks). (**A**) Compound 8 is bound to the protease active site of the NS3 protein; (**B**) RK-0404678 is bound to the RNA binding site of the NS5 RdRp, close to the priming loop; (**C**) HeE1-2Tyr is bound to the RNA template tunnel, between the fingers subdomain and the priming loop; (**D**) Ribavirin 5′-triphosphate is bound to the GTP binding site of the NS5 MTase; (**E**) Sinefungin is bound to the SAM binding site of the NS5 MTase. All structure representations were made in PyMOL.

Overall, advances in structure-guided approaches used in the design of potent allosteric inhibitors show promising potential for the development of more potent inhibitors that can target DENV. Representative crystal structures of DENV NS5 solved with some of the potent inhibitors are shown in Figure 4.

### 3.2. Other Dengue Antiviral Targets

Other antiviral targets evaluated for antiviral activity against DENV includes the NS1 protein, the envelope protein, and the viral capsid.

#### 3.2.1. NS1

NS1 is a nonstructural protein of approximately 46–55 kDa depending on its glycosylation state. It is found in different cellular locations and occurs in multiple oligomeric forms. NS1 is also present in the ER-resident form, the membrane-anchored form, and the secreted form. It is first produced as a soluble monomer, and then associates with the membrane after dimerization in the ER lumen [133]. Intracellular NS1 is known to participate in early viral RNA replication. It is also transported to the surface of the cell where it either associates with the membrane or is secreted as a soluble hexamer [134]. The specific function of NS1 in the viral replication cycle is yet to be fully understood, but studies have shown that it is highly immunogenic making it a potential candidate for vaccine target and is being used as a diagnostic marker in confirming dengue infection [133,135,136].

#### 3.2.2. Envelope Protein

The DENV envelope protein, a 53 kDa dimer is the major component of the virion surface. In its dimeric form, the E protein becomes adequate for binding to the cell surface, fusion, and viral entry into host cells [96,137]. The E protein has approximately 40% sequence homology among flaviviruses [135].

Many heterocyclic compounds identified in silico, were found to have activity against DENV E protein. NITD-448 compound was found to inhibit DENV E protein-mediated membrane fusion. Compounds D02, D04, and D05 were found to inhibit maturation or viral host cell entry due to their binding to the E protein [94,138].

#### 3.2.3. Viral Capsid

The DENV viral capsid is approximately 11 kDa and interacts with the genomic RNA to form the nucleocapsid, which is essential for dimerization in viral assembly. The capsid protein also contains an internal hydrophobic sequence required for membrane association between the virus and the host cell receptor [96].

The capsid is also known to be a potential target for antiviral agents. Compound ST-148 was identified as a DENV capsid inhibitor. It was shown to block the cytopathic effect caused by DENV and was found to be effective against all four DENV serotypes [139].

### 3.3. DENV Small Molecule Inhibitors in Clinical Trials

Antiviral research efforts have targeted both structural and NS DENV proteins, however specific focus has been put on the NS3 and NS5 proteins, because of their multifunctional nature [66,140]. Unfortunately, no antiviral agents developed specifically to target DENV have entered clinical trials to date [45]. A nucleoside analogue, balapiravir was clinically investigated as a DENV NS5 inhibitor. Balapiravir was originally developed as an antiviral agent against hepatitis C virus (HCV) and was evaluated in a phase 1 clinical trial as a short-course antiviral against DENV [43]. However, it failed to meet the efficacy endpoint and was found to poorly permeate target cells, resulting in termination of clinical development against dengue infection [141].

### 3.4. Alternative Therapeutic Approaches

Since there is no specific treatment for dengue currently available, dengue patients are usually provided with supportive care including bed rest, fluid replacement therapy, analgesics, and antipyretics to relieve the fever [5]. There is currently no effective vaccine available to prevent the disease, and vector control strategies have been mostly ineffective and expensive [142,143]. A study was recently conducted in Indonesia to assess the efficacy of Wolbachia-infected *Aedes aegypti* mosquitoes for the control of dengue. Interestingly, the study showed that the deployment of the infected mosquitoes into wild-type *Aedes aegypti* mosquito populations effectively reduced the incidence of symptomatic dengue cases and resulted in fewer dengue hospitalizations [144]. The results from this study show the potential of the use of genetically modified mosquitoes as a dengue vector control strategy.

None of the small molecule inhibitors developed against dengue has advanced beyond early clinical trials which shows the need for continued efforts in the design of novel therapeutic approaches against dengue. Nucleic acid-based therapies have been proposed as an alternative approach, which can inhibit gene expression and act as antivirals against dengue [145]. RNA interference (RNAi) has been employed as a therapeutic approach in tumors, metabolic disorders, and several infectious diseases, and can potentially be applied to treat dengue. This approach has been proposed to protect the host cell from viral infections by degradation of viral RNA although flaviviral RNA has been shown to be resistant to RNAi because replication occurs in assembled ER membrane packets. RNA decoys, like phosphorodiamidate morpholino oligomers (PMOs), which target the translation initiation site (5′UTR) of DENV RNA are promising therapeutics but are yet to be tested in animal models [5].

Advances in molecular and structural virology have propelled drug discovery efforts with increasing structural information being obtained on the dengue virus and nonstructural proteins crucial for the viral life cycle [45]. High-resolution structures determined by cryo-electron microscopy (Cryo-EM), nuclear magnetic resonance (NMR) spectroscopy, and X-ray crystallography are now being used in combination with in-silico approaches and infectious clone methods to identify new targets for DENV [66,146].

The complex nature of the human immune response to DENV, especially the antibody-dependent enhancement phenomenon, makes it more difficult to develop potent and effective therapeutics. High-throughput sequencing approaches and advancements in computational analysis of complex data has however provided the means for better assessment and analysis of the DENV immune response [44]. Recent studies have shown that machine learning models, complex statistical analyses and visualizations can be applied to answering questions about ADE, and potentially identifying novel therapeutics against dengue [147,148,149].

## 4. Conclusions

Currently, the overall efforts to develop therapeutic avenues against dengue virus have not stemmed the increasing trends of global disease burden and geographical expansion. Thus far, the major axis of therapeutic efforts against DENV have largely centered on the development of vaccines. These efforts have advanced to late clinical trials although with limited end results. Conversely, the research efforts to develop antivirals targeting DENV have been mostly underwhelming with most development efforts in the preclinical stages. Multiple research efforts are currently ongoing in the development of antivirals against dengue, especially those against NS3 and NS5 proteins. The advancement of antiviral research focused on dengue NS5 as a target is, however, still limited by its low structural and mechanistic characterization, when compared to the viral RNA or DNA polymerase from other viruses with more developed therapeutic efforts such as HIV and HCV. More in-depth characterization of the structure of the dengue NS5 protein and mechanistic insights into how it performs its multiple functions will strengthen drug discovery efforts geared towards NS5 as a target against the dengue virus.

## Figures and Tables

**Table 1:** Small Molecule Inhibitors against dengue NS3 and NS5.

Target	Function	PDB ID	Compound Name	K_i_ or K_D_ (μM)	IC_50_ (μM)	References
NS5	MTase	3P8Z	Compound **10**	0.82 (N7), 0.17 (2′-O)	na	[73]
		1R6A	Ribavirin 5′-triphosphate	55 *	100	[74]
		5EHI	BF287	na	452	[75]
		5EKX	NB2E11	na	>1000	[75]
		5EHG	BF341	na	369	[75]
		5EIF	NB2C3	na	>1000	[75]
		5EC8	BF175	na	na	[75]
		5EIW	NB3C2	na	>1000	[75]
		5E9Q	BF174	na	na	[75]
		4R8S	Sinefungin	0.136 *	0.03 (N7), 0.04 (2′-O)	[76,77,78]
		na	1-TP	22	8.4	[79]
		na	2-TP	na	1.1	[79]
	RdRp	5K5M	Compound **27**	na	0.173	[80]
		5I3P	Compound **27**	na	0.048	[80]
		5I3Q	Compound **29**	na	0.016	[80]
		5IQ6	HeE1-2Tyr	1.96	1.5	[81]
		5HMW	Compound **5**	154 *	177	[82]
		5HMY	Compound **15**	1.4 *	1.7	[82]
		6IZX	RK-0404678	na	201	[83]
		5HMX	Compound **10**	28 *	15	[82]
		5HN0	Compound **4**	>200 *	769	[82]
		5HMZ	Compound **23**	0.12 *	0.34	[82]
		3VWS	NITD-107	225 *	113	[84]
		5F3Z	PC-79-SH52	29 *	140	[85]
		5F3T	JF-31-MG46	210	730	[85]
		5F41	FD-83-KI26	67 *	210	[85]
		6IZZ	RK-0404678	na	287	[83]
NS3	Protease (NS2B-NS3)	4M9T	DTNB	na	na	[86]
		3U1J	Aprotinin	0.026	na	[87,88]
		6MO1	Compound **8**	na	0.29	[89]
		6MO2	Compound **9**	na	0.59	[89]

IC_50_—Half maximal inhibitory concentration; K_i_—inhibition constant; K_D_—dissociation constant; * indicates the value shown is a K_D_; PDB—protein data bank.

## Data Availability

Not applicable.

## References

[1] Mitra A.K., Mawson A.R. (2017). Neglected Tropical Diseases: Epidemiology and Global Burden. Trop. Med. Infect. Dis..

[2] Kraemer M.U., Sinka M.E., Duda K.A., Mylne A.Q., Shearer F.M., Barker C.M., Moore C.G., Carvalho R.G., Coelho G.E., Van Bortel W. (2015). The global distribution of the arbovirus vectors Aedes aegypti and Ae. albopictus. Elife.

[3] Bhatt S., Gething P.W., Brady O.J., Messina J.P., Farlow A.W., Moyes C.L., Drake J.M., Brownstein J.S., Hoen A.G., Sankoh O. (2013). The global distribution and burden of dengue. Nature.

[4] Guzman M.G., Harris E. (2015). Dengue. Lancet.

[5] Back A.T., Lundkvist A. (2013). Dengue viruses—An overview. Infect. Ecol. Epidemiol..

[6] Wilder-Smith A., Ooi E.E., Horstick O., Wills B. (2019). Dengue. Lancet.

[7] Rocklöv J., Quam M.B., Sudre B., German M., Kraemer M.U.G., Brady O., Bogoch I.I., Liu-Helmersson J., Wilder-Smith A., Semenza J.C. (2016). Assessing Seasonal Risks for the Introduction and Mosquito-borne Spread of Zika Virus in Europe. EBioMedicine.

[8] Guzman M.G., Gubler D.J., Izquierdo A., Martinez E., Halstead S.B. (2016). Dengue infection. Nat. Rev. Dis. Primers.

[9] Messina J.P., Brady O.J., Scott T.W., Zou C., Pigott D.M., Duda K.A., Bhatt S., Katzelnick L., Howes R.E., Battle K.E. (2014). Global spread of dengue virus types: Mapping the 70 year history. Trends Microbiol..

[10] WHO Dengue and Severe Dengue. https://www.who.int/news-room/fact-sheets/detail/dengue-and-severe-dengue.

[11] Hadinegoro S.R., Arredondo-García J.L., Capeding M.R., Deseda C., Chotpitayasunondh T., Dietze R., Muhammad Ismail H.I., Reynales H., Limkittikul K., Rivera-Medina D.M. (2015). Efficacy and Long-Term Safety of a Dengue Vaccine in Regions of Endemic Disease. N. Engl. J. Med..

[12] Gubler D.J. (1998). Dengue and dengue hemorrhagic fever. Clin. Microbiol. Rev..

[13] Endy T.P., Anderson K.B., Nisalak A., Yoon I.K., Green S., Rothman A.L., Thomas S.J., Jarman R.G., Libraty D.H., Gibbons R.V. (2011). Determinants of inapparent and symptomatic dengue infection in a prospective study of primary school children in Kamphaeng Phet, Thailand. PLoS Negl. Trop. Dis..

[14] Amarasinghe A., Kuritsk J.N., Letson G.W., Margolis H.S. (2011). Dengue virus infection in Africa. Emerg. Infect. Dis..

[15] Gubler D.J. (2011). Dengue, Urbanization and Globalization: The Unholy Trinity of the 21(st) Century. Trop. Med. Health.

[16] Murray C.J., Vos T., Lozano R., Naghavi M., Flaxman A.D., Michaud C., Ezzati M., Shibuya K., Salomon J.A., Abdalla S. (2012). Disability-adjusted life years (DALYs) for 291 diseases and injuries in 21 regions, 1990–2010: A systematic analysis for the Global Burden of Disease Study 2010. Lancet.

[17] Shepard D.S., Undurraga E.A., Halasa Y.A., Stanaway J.D. (2016). The global economic burden of dengue: A systematic analysis. Lancet Infect. Dis..

[18] Shepard D.S., Undurraga E.A., Betancourt-Cravioto M., Guzmán M.G., Halstead S.B., Harris E., Mudin R.N., Murray K.O., Tapia-Conyer R., Gubler D.J. (2014). Approaches to refining estimates of global burden and economics of dengue. PLoS Negl. Trop. Dis..

[19] Messina J.P., Brady O.J., Golding N., Kraemer M.U.G., Wint G.R.W., Ray S.E., Pigott D.M., Shearer F.M., Johnson K., Earl L. (2019). The current and future global distribution and population at risk of dengue. Nat. Microbiol..

[20] Martina B.E., Koraka P., Osterhaus A.D. (2009). Dengue virus pathogenesis: An integrated view. Clin. Microbiol. Rev..

[21] Joob B., Wiwanitkit V. (2016). Fifth serotype of dengue virus: What we should prepare for?. Med. J. Armed Forces India.

[22] Mustafa M.S., Rasotgi V., Jain S., Gupta V. (2015). Discovery of fifth serotype of dengue virus (DENV-5): A new public health dilemma in dengue control. Med. J. Armed Forces India.

[23] Montoya M., Gresh L., Mercado J.C., Williams K.L., Vargas M.J., Gutierrez G., Kuan G., Gordon A., Balmaseda A., Harris E. (2013). Symptomatic versus inapparent outcome in repeat dengue virus infections is influenced by the time interval between infections and study year. PLoS Negl. Trop. Dis..

[24] Snow G.E., Haaland B., Ooi E.E., Gubler D.J. (2014). Review article: Research on dengue during World War II revisited. Am. J. Trop. Med. Hyg..

[25] Guzman M.G., Halstead S.B., Artsob H., Buchy P., Farrar J., Gubler D.J., Hunsperger E., Kroeger A., Margolis H.S., Martínez E. (2010). Dengue: A continuing global threat. Nat. Rev. Microbiol..

[26] Halstead S.B., Nimmannitya S., Cohen S.N. (1970). Observations related to pathogenesis of dengue hemorrhagic fever. IV. Relation of disease severity to antibody response and virus recovered. Yale J. Biol. Med..

[27] Halstead S.B., O’Rourke E.J. (1977). Dengue viruses and mononuclear phagocytes. I. Infection enhancement by non-neutralizing antibody. J. Exp. Med..

[28] Chan K.R., Ong E.Z., Tan H.C., Zhang S.L., Zhang Q., Tang K.F., Kaliaperumal N., Lim A.P., Hibberd M.L., Chan S.H. (2014). Leukocyte immunoglobulin-like receptor B1 is critical for antibody-dependent dengue. Proc. Natl. Acad. Sci. USA.

[29] Ong E.Z., Zhang S.L., Tan H.C., Gan E.S., Chan K.R., Ooi E.E. (2017). Dengue virus compartmentalization during antibody-enhanced infection. Sci. Rep..

[30] Rothman A.L. (2011). Immunity to dengue virus: A tale of original antigenic sin and tropical cytokine storms. Nat. Rev. Immunol..

[31] Yacoub S., Wertheim H., Simmons C.P., Screaton G., Wills B. (2015). Microvascular and endothelial function for risk prediction in dengue: An observational study. Lancet.

[32] Messer W.B., Yount B., Hacker K.E., Donaldson E.F., Huynh J.P., de Silva A.M., Baric R.S. (2012). Development and characterization of a reverse genetic system for studying dengue virus serotype 3 strain variation and neutralization. PLoS Negl. Trop. Dis..

[33] Bara J.J., Clark T.M., Remold S.K. (2013). Susceptibility of larval Aedes aegypti and Aedes albopictus (Diptera: Culicidae) to dengue virus. J. Med. Entomol..

[34] Rico-Hesse R. (2003). Microevolution and virulence of dengue viruses. Adv. Virus Res..

[35] Halstead S.B. (2008). Dengue virus-mosquito interactions. Annu. Rev. Entomol..

[36] Gubler D.J., Reed D., Rosen L., Hitchcock J.R. (1978). Epidemiologic, clinical, and virologic observations on dengue in the Kingdom of Tonga. Am. J. Trop. Med. Hyg..

[37] Rosen L. (1977). The Emperor’s New Clothes revisited, or reflections on the pathogenesis of dengue hemorrhagic fever. Am. J. Trop. Med. Hyg..

[38] Godói I.P., Lemos L.L., de Araújo V.E., Bonoto B.C., Godman B., Guerra Júnior A.A. (2017). CYD-TDV dengue vaccine: Systematic review and meta-analysis of efficacy, immunogenicity and safety. J. Comp. Eff. Res..

[39] Osorio J.E., Wallace D., Stinchcomb D.T. (2016). A recombinant, chimeric tetravalent dengue vaccine candidate based on a dengue virus serotype 2 backbone. Expert Rev. Vaccines.

[40] Whitehead S.S. (2016). Development of TV003/TV005, a single dose, highly immunogenic live attenuated dengue vaccine; what makes this vaccine different from the Sanofi-Pasteur CYD™ vaccine?. Expert Rev. Vaccines.

[41] Torresi J., Ebert G., Pellegrini M. (2017). Vaccines licensed and in clinical trials for the prevention of dengue. Hum. Vaccin. Immunother..

[42] Low J.G., Sung C., Wijaya L., Wei Y., Rathore A.P.S., Watanabe S., Tan B.H., Toh L., Chua L.T., Hou Y. (2014). Efficacy and safety of celgosivir in patients with dengue fever (CELADEN): A phase 1b, randomised, double-blind, placebo-controlled, proof-of-concept trial. Lancet Infect. Dis..

[43] Nguyen N.M., Tran C.N., Phung L.K., Duong K.T., Huynh H.e.A., Farrar J., Nguyen Q.T., Tran H.T., Nguyen C.V., Merson L. (2013). A randomized, double-blind placebo controlled trial of balapiravir, a polymerase inhibitor, in adult dengue patients. J. Infect. Dis..

[44] Natali E.N., Babrak L.M., Miho E. (2021). Prospective Artificial Intelligence to Dissect the Dengue Immune Response and Discover Therapeutics. Front. Immunol..

[45] Low J.G., Ooi E.E., Vasudevan S.G. (2017). Current Status of Dengue Therapeutics Research and Development. J. Infect. Dis..

[46] Modis Y., Ogata S., Clements D., Harrison S.C. (2003). A ligand-binding pocket in the dengue virus envelope glycoprotein. Proc. Natl. Acad. Sci. USA.

[47] Pang X., Zhang M., Dayton A.I. (2001). Development of dengue virus replicons expressing HIV-1 gp120 and other heterologous genes: A potential future tool for dual vaccination against dengue virus and HIV. BMC Microbiol..

[48] Pang X., Zhang M., Dayton A.I. (2001). Development of Dengue virus type 2 replicons capable of prolonged expression in host cells. BMC Microbiol..

[49] Alvarez D.E., Lodeiro M.F., Filomatori C.V., Fucito S., Mondotte J.A., Gamarnik A.V. (2006). Structural and functional analysis of dengue virus RNA. Novartis. Found. Symp..

[50] Gamarnik A., Hanley K.A., Weaver S.C. (2010). Role of the dengue virus 5′ and 3′ untranslated regions in viral replication. Frontiers in Dengue Virus Research.

[51] Padmanabhan R., Strongin A.Y., Hanley K.A., Weaver S.C. (2010). Translation and processing of the dengue virus polyprotein. Frontiers in Dengue Virus Research.

[52] Yu L., Nomaguchi M., Padmanabhan R., Markoff L. (2008). Specific requirements for elements of the 5′ and 3′ terminal regions in flavivirus RNA synthesis and viral replication. Virology.

[53] Heinz F.X., Allison S.L. (2003). Flavivirus structure and membrane fusion. Adv. Virus Res..

[54] Chen Y., Maguire T., Hileman R.E., Fromm J.R., Esko J.D., Linhardt R.J., Marks R.M. (1997). Dengue virus infectivity depends on envelope protein binding to target cell heparan sulfate. Nat. Med..

[55] Tassaneetrithep B., Burgess T.H., Granelli-Piperno A., Trumpfheller C., Finke J., Sun W., Eller M.A., Pattanapanyasat K., Sarasombath S., Birx D.L. (2003). DC-SIGN (CD209) mediates dengue virus infection of human dendritic cells. J. Exp. Med..

[56] Miller J.L., de Wet B.J., de Wet B.J., Martinez-Pomares L., Radcliffe C.M., Dwek R.A., Rudd P.M., Gordon S. (2008). The mannose receptor mediates dengue virus infection of macrophages. PLoS Pathog..

[57] Chen S.T., Lin Y.L., Huang M.T., Wu M.F., Cheng S.C., Lei H.Y., Lee C.K., Chiou T.W., Wong C.H., Hsieh S.L. (2008). CLEC5A is critical for dengue-virus-induced lethal disease. Nature.

[58] Watson A.A., Lebedev A.A., Hall B.A., Fenton-May A.E., Vagin A.A., Dejnirattisai W., Felce J., Mongkolsapaya J., Palma A.S., Liu Y. (2011). Structural flexibility of the macrophage dengue virus receptor CLEC5A: Implications for ligand binding and signaling. J. Biol. Chem..

[59] Clyde K., Kyle J.L., Harris E. (2006). Recent advances in deciphering viral and host determinants of dengue virus replication and pathogenesis. J. Virol..

[60] Stadler K., Allison S.L., Schalich J., Heinz F.X. (1997). Proteolytic activation of tick-borne encephalitis virus by furin. J. Virol..

[61] Guirakhoo F., Heinz F.X., Mandl C.W., Holzmann H., Kunz C. (1991). Fusion activity of flaviviruses: Comparison of mature and immature (prM-containing) tick-borne encephalitis virions. J. Gen. Virol..

[62] Guirakhoo F., Bolin R.A., Roehrig J.T. (1992). The Murray Valley encephalitis virus prM protein confers acid resistance to virus particles and alters the expression of epitopes within the R2 domain of E glycoprotein. Virology.

[63] Zhang Y., Corver J., Chipman P.R., Zhang W., Pletnev S.V., Sedlak D., Baker T.S., Strauss J.H., Kuhn R.J., Rossmann M.G. (2003). Structures of immature flavivirus particles. EMBO J..

[64] Garcia L.L., Padilla L., Castano J.C. (2017). Inhibitors compounds of the flavivirus replication process. Virol. J..

[65] Apte-Sengupta S., Sirohi D., Kuhn R.J. (2014). Coupling of replication and assembly in flaviviruses. Curr. Opin. Virol..

[66] Luo D., Vasudevan S.G., Lescar J. (2015). The flavivirus NS2B-NS3 protease-helicase as a target for antiviral drug development. Antiviral Res..

[67] Zou G., Chen Y.L., Dong H., Lim C.C., Yap L.J., Yau Y.H., Shochat S.G., Lescar J., Shi P.Y. (2011). Functional analysis of two cavities in flavivirus NS5 polymerase. J. Biol. Chem..

[68] Yang C.C., Hu H.S., Wu R.H., Wu S.H., Lee S.J., Jiaang W.T., Chern J.H., Huang Z.S., Wu H.N., Chang C.M. (2014). A novel dengue virus inhibitor, BP13944, discovered by high-throughput screening with dengue virus replicon cells selects for resistance in the viral NS2B/NS3 protease. Antimicrob. Agents Chemother..

[69] Steuer C., Gege C., Fischl W., Heinonen K.H., Bartenschlager R., Klein C.D. (2011). Synthesis and biological evaluation of α-ketoamides as inhibitors of the Dengue virus protease with antiviral activity in cell-culture. Bioorg. Med. Chem..

[70] Tomlinson S.M., Watowich S.J. (2011). Anthracene-based inhibitors of dengue virus NS2B-NS3 protease. Antiviral Res..

[71] Bollati M., Alvarez K., Assenberg R., Baronti C., Canard B., Cook S., Coutard B., Decroly E., de Lamballerie X., Gould E.A. (2010). Structure and functionality in flavivirus NS-proteins: Perspectives for drug design. Antiviral Res..

[72] Noble C.G., Chen Y.L., Dong H., Gu F., Lim S.P., Schul W., Wang Q.Y., Shi P.Y. (2010). Strategies for development of Dengue virus inhibitors. Antiviral Res..

[73] Lim S.P., Sonntag L.S., Noble C., Nilar S.H., Ng R.H., Zou G., Monaghan P., Chung K.Y., Dong H., Liu B. (2011). Small molecule inhibitors that selectively block dengue virus methyltransferase. J. Biol. Chem..

[74] Benarroch D., Egloff M.P., Mulard L., Guerreiro C., Romette J.L., Canard B. (2004). A structural basis for the inhibition of the NS5 dengue virus mRNA 2’-O-methyltransferase domain by ribavirin 5’-triphosphate. J. Biol. Chem..

[75] Benmansour F., Trist I., Coutard B., Decroly E., Querat G., Brancale A., Barral K. (2017). Discovery of novel dengue virus NS5 methyltransferase non-nucleoside inhibitors by fragment-based drug design. Eur. J. Med. Chem..

[76] Noble C.G., Li S.H., Dong H., Chew S.H., Shi P.Y. (2014). Crystal structure of dengue virus methyltransferase without S-adenosyl-L-methionine. Antiviral Res..

[77] Brecher M.B., Li Z., Zhang J., Chen H., Lin Q., Liu B., Li H. (2015). Refolding of a fully functional flavivirus methyltransferase revealed that S-adenosyl methionine but not S-adenosyl homocysteine is copurified with flavivirus methyltransferase. Protein Sci..

[78] Chung K.Y., Dong H., Chao A.T., Shi P.Y., Lescar J., Lim S.P. (2010). Higher catalytic efficiency of N-7-methylation is responsible for processive N-7 and 2′-O methyltransferase activity in dengue virus. Virology.

[79] Thames J.E., Waters C.D., Valle C., Bassetto M., Aouadi W., Martin B., Selisko B., Falat A., Coutard B., Brancale A. (2020). Synthesis and biological evaluation of novel flexible nucleoside analogues that inhibit flavivirus replication in vitro. Bioorg. Med. Chem..

[80] Lim S.P., Noble C.G., Seh C.C., Soh T.S., El Sahili A., Chan G.K., Lescar J., Arora R., Benson T., Nilar S. (2016). Potent Allosteric Dengue Virus NS5 Polymerase Inhibitors: Mechanism of Action and Resistance Profiling. PLoS Pathog..

[81] Tarantino D., Cannalire R., Mastrangelo E., Croci R., Querat G., Barreca M.L., Bolognesi M., Manfroni G., Cecchetti V., Milani M. (2016). Targeting flavivirus RNA dependent RNA polymerase through a pyridobenzothiazole inhibitor. Antiviral Res..

[82] Yokokawa F., Nilar S., Noble C.G., Lim S.P., Rao R., Tania S., Wang G., Lee G., Hunziker J., Karuna R. (2016). Discovery of Potent Non-Nucleoside Inhibitors of Dengue Viral RNA-Dependent RNA Polymerase from a Fragment Hit Using Structure-Based Drug Design. J. Med. Chem..

[83] Shimizu H., Saito A., Mikuni J., Nakayama E.E., Koyama H., Honma T., Shirouzu M., Sekine S.I., Shioda T. (2019). Discovery of a small molecule inhibitor targeting dengue virus NS5 RNA-dependent RNA polymerase. PLoS Negl. Trop. Dis..

[84] Noble C.G., Lim S.P., Chen Y.L., Liew C.W., Yap L., Lescar J., Shi P.Y. (2013). Conformational flexibility of the Dengue virus RNA-dependent RNA polymerase revealed by a complex with an inhibitor. J. Virol..

[85] Noble C.G., Lim S.P., Arora R., Yokokawa F., Nilar S., Seh C.C., Wright S.K., Benson T.E., Smith P.W., Shi P.Y. (2016). A Conserved Pocket in the Dengue Virus Polymerase Identified through Fragment-based Screening. J. Biol. Chem..

[86] Yildiz M., Ghosh S., Bell J.A., Sherman W., Hardy J.A. (2013). Allosteric inhibition of the NS2B-NS3 protease from dengue virus. ACS Chem. Biol..

[87] Noble C.G., Seh C.C., Chao A.T., Shi P.Y. (2012). Ligand-bound structures of the dengue virus protease reveal the active conformation. J. Virol..

[88] Mueller N.H., Yon C., Ganesh V.K., Padmanabhan R. (2007). Characterization of the West Nile virus protease substrate specificity and inhibitors. Int. J. Biochem. Cell Biol..

[89] Yao Y., Huo T., Lin Y.L., Nie S., Wu F., Hua Y., Wu J., Kneubehl A.R., Vogt M.B., Rico-Hesse R. (2019). Discovery, X-ray Crystallography and Antiviral Activity of Allosteric Inhibitors of Flavivirus NS2B-NS3 Protease. J. Am. Chem. Soc..

[90] Luo D., Xu T., Watson R.P., Scherer-Becker D., Sampath A., Jahnke W., Yeong S.S., Wang C.H., Lim S.P., Strongin A. (2008). Insights into RNA unwinding and ATP hydrolysis by the flavivirus NS3 protein. EMBO J..

[91] Sweeney N.L., Hanson A.M., Mukherjee S., Ndjomou J., Geiss B.J., Steel J.J., Frankowski K.J., Li K., Schoenen F.J., Frick D.N. (2015). Benzothiazole and Pyrrolone Flavivirus Inhibitors Targeting the Viral Helicase. ACS Infect. Dis..

[92] Basavannacharya C., Vasudevan S.G. (2014). Suramin inhibits helicase activity of NS3 protein of dengue virus in a fluorescence-based high throughput assay format. Biochem. Biophys. Res. Commun..

[93] Xie X., Zou J., Wang Q.Y., Shi P.Y. (2015). Targeting dengue virus NS4B protein for drug discovery. Antiviral Res..

[94] Lim S.P., Wang Q.Y., Noble C.G., Chen Y.L., Dong H., Zou B., Yokokawa F., Nilar S., Smith P., Beer D. (2013). Ten years of dengue drug discovery: Progress and prospects. Antiviral Res..

[95] van Cleef K.W., Overheul G.J., Thomassen M.C., Kaptein S.J., Davidson A.D., Jacobs M., Neyts J., van Kuppeveld F.J., van Rij R.P. (2013). Identification of a new dengue virus inhibitor that targets the viral NS4B protein and restricts genomic RNA replication. Antiviral Res..

[96] Sampath A., Padmanabhan R. (2009). Molecular targets for flavivirus drug discovery. Antiviral Res..

[97] Yap T.L., Xu T., Chen Y.L., Malet H., Egloff M.P., Canard B., Vasudevan S.G., Lescar J. (2007). Crystal structure of the dengue virus RNA-dependent RNA polymerase catalytic domain at 1.85-angstrom resolution. J. Virol..

[98] Malet H., Massé N., Selisko B., Romette J.L., Alvarez K., Guillemot J.C., Tolou H., Yap T.L., Vasudevan S., Lescar J. (2008). The flavivirus polymerase as a target for drug discovery. Antiviral Res..

[99] Najera I. (2013). Resistance to HCV nucleoside analogue inhibitors of hepatitis C virus RNA-dependent RNA polymerase. Curr. Opin. Virol..

[100] Ferrer-Orta C., Arias A., Escarmís C., Verdaguer N. (2006). A comparison of viral RNA-dependent RNA polymerases. Curr. Opin. Struct. Biol..

[101] Zhao Y., Soh T.S., Zheng J., Chan K.W., Phoo W.W., Lee C.C., Tay M.Y., Swaminathan K., Cornvik T.C., Lim S.P. (2015). A crystal structure of the Dengue virus NS5 protein reveals a novel inter-domain interface essential for protein flexibility and virus replication. PLoS Pathog..

[102] Bujalowski P.J., Bujalowski W., Choi K.H. (2017). Interactions between the Dengue Virus Polymerase NS5 and Stem-Loop A. J. Virol..

[103] Wang B., Thurmond S., Zhou K., Sánchez-Aparicio M.T., Fang J., Lu J., Gao L., Ren W., Cui Y., Veit E.C. (2020). Structural basis for STAT2 suppression by flavivirus NS5. Nat. Struct. Mol. Biol..

[104] Lim S.P., Noble C.G., Shi P.Y. (2015). The dengue virus NS5 protein as a target for drug discovery. Antiviral Res..

[105] Chang J., Schul W., Butters T.D., Yip A., Liu B., Goh A., Lakshminarayana S.B., Alonzi D., Reinkensmeier G., Pan X. (2011). Combination of α-glucosidase inhibitor and ribavirin for the treatment of dengue virus infection in vitro and in vivo. Antiviral Res..

[106] Malinoski F.J., Hasty S.E., Ussery M.A., Dalrymple J.M. (1990). Prophylactic ribavirin treatment of dengue type 1 infection in rhesus monkeys. Antiviral Res..

[107] Yates M.K., Raje M.R., Chatterjee P., Spiropoulou C.F., Bavari S., Flint M., Soloveva V., Seley-Radtke K.L. (2017). Flex-nucleoside analogues—Novel therapeutics against filoviruses. Bioorg. Med. Chem. Lett..

[108] Peters H.L., Jochmans D., de Wilde A.H., Posthuma C.C., Snijder E.J., Neyts J., Seley-Radtke K.L. (2015). Design, synthesis and evaluation of a series of acyclic fleximer nucleoside analogues with anti-coronavirus activity. Bioorg. Med. Chem. Lett..

[109] Seley K.L., Zhang L., Hagos A., Quirk S. (2002). "Fleximers". Design and synthesis of a new class of novel shape-modified nucleosides(1). J. Org. Chem..

[110] Seley K.L., Quirk S., Salim S., Zhang L., Hagos A. (2003). Unexpected inhibition of S-adenosyl-L-homocysteine hydrolase by a guanosine nucleoside. Bioorg. Med. Chem. Lett..

[111] Quirk S., Seley K.L. (2005). Substrate discrimination by the human GTP fucose pyrophosphorylase. Biochemistry.

[112] Eyer L., Nencka R., de Clercq E., Seley-Radtke K., Růžek D. (2018). Nucleoside analogs as a rich source of antiviral agents active against arthropod-borne flaviviruses. Antivir. Chem. Chemother..

[113] Ackermann M., Padmanabhan R. (2001). De novo synthesis of RNA by the dengue virus RNA-dependent RNA polymerase exhibits temperature dependence at the initiation but not elongation phase. J. Biol. Chem..

[114] Nomaguchi M., Teramoto T., Yu L., Markoff L., Padmanabhan R. (2004). Requirements for West Nile virus (−)- and (+)-strand subgenomic RNA synthesis in vitro by the viral RNA-dependent RNA polymerase expressed in Escherichia coli. J. Biol. Chem..

[115] Jordheim L.P., Durantel D., Zoulim F., Dumontet C. (2013). Advances in the development of nucleoside and nucleotide analogues for cancer and viral diseases. Nat. Rev. Drug Discov..

[116] Benhamou Y., Tubiana R., Thibault V. (2003). Tenofovir disoproxil fumarate in patients with HIV and lamivudine-resistant hepatitis B virus. N. Engl. J. Med..

[117] Ray A.S., Fordyce M.W., Hitchcock M.J. (2016). Tenofovir alafenamide: A novel prodrug of tenofovir for the treatment of Human Immunodeficiency Virus. Antiviral Res..

[118] Stedman C. (2014). Sofosbuvir, a NS5B polymerase inhibitor in the treatment of hepatitis C: A review of its clinical potential. Therap. Adv. Gastroenterol..

[119] Huang Y.S., Chang S.Y., Sheng W.H., Sun H.Y., Lee K.Y., Chuang Y.C., Su Y.C., Liu W.C., Hung C.C., Chang S.C. (2016). Virological Response to Tenofovir Disoproxil Fumarate in HIV-Positive Patients with Lamivudine-Resistant Hepatitis B Virus Coinfection in an Area Hyperendemic for Hepatitis B Virus Infection. PLoS ONE.

[120] Lam Y.F., Seto W.K., Wong D., Cheung K.S., Fung J., Mak L.Y., Yuen J., Chong C.K., Lai C.L., Yuen M.F. (2017). Seven-Year Treatment Outcome of Entecavir in a Real-World Cohort: Effects on Clinical Parameters, HBsAg and HBcrAg Levels. Clin. Transl. Gastroenterol..

[121] Deval J., Symons J.A., Beigelman L. (2014). Inhibition of viral RNA polymerases by nucleoside and nucleotide analogs: Therapeutic applications against positive-strand RNA viruses beyond hepatitis C virus. Curr. Opin. Virol..

[122] Behnam M.A., Nitsche C., Boldescu V., Klein C.D. (2016). The Medicinal Chemistry of Dengue Virus. J. Med. Chem..

[123] Boldescu V., Behnam M.A.M., Vasilakis N., Klein C.D. (2017). Broad-spectrum agents for flaviviral infections: Dengue, Zika and beyond. Nat. Rev. Drug Discov..

[124] Choi K.H., Rossmann M.G. (2009). RNA-dependent RNA polymerases from Flaviviridae. Curr. Opin. Struct. Biol..

[125] De Clercq E., Neyts J. (2009). Antiviral agents acting as DNA or RNA chain terminators. Handb. Exp. Pharmacol..

[126] De Clercq E. (2004). Antivirals and antiviral strategies. Nat. Rev. Microbiol..

[127] Cihlar T., Ray A.S. (2010). Nucleoside and nucleotide HIV reverse transcriptase inhibitors: 25 years after zidovudine. Antiviral Res..

[128] De Clercq E. (2005). Recent highlights in the development of new antiviral drugs. Curr. Opin. Microbiol..

[129] Bartholomeusz A., Tomlinson E., Wright P.J., Birch C., Locarnini S., Weigold H., Marcuccio S., Holan G. (1994). Use of a flavivirus RNA-dependent RNA polymerase assay to investigate the antiviral activity of selected compounds. Antiviral Res..

[130] Herve M., Sinoussi-Barre F., Chermann J.C., Herve G., Jasmin C. (1983). Correlation between structure of polyoxotungstates and their inhibitory activity on polymerases. Biochem. Biophys. Res. Commun..

[131] Caillet-Saguy C., Simister P.C., Bressanelli S. (2011). An objective assessment of conformational variability in complexes of hepatitis C virus polymerase with non-nucleoside inhibitors. J. Mol. Biol..

[132] Lim S.P., Noble C.G., Nilar S., Shi P.Y., Yokokawa F. (2018). Discovery of Potent Non-nucleoside Inhibitors of Dengue Viral RNA-Dependent RNA Polymerase from Fragment Screening and Structure-Guided Design. Adv. Exp. Med. Biol..

[133] Muller D.A., Young P.R. (2013). The flavivirus NS1 protein: Molecular and structural biology, immunology, role in pathogenesis and application as a diagnostic biomarker. Antiviral Res..

[134] Mackenzie J.M., Jones M.K., Young P.R. (1996). Immunolocalization of the dengue virus nonstructural glycoprotein NS1 suggests a role in viral RNA replication. Virology.

[135] Heinz F.X., Stiasny K. (2012). Flaviviruses and flavivirus vaccines. Vaccine.

[136] Libraty D.H., Young P.R., Pickering D., Endy T.P., Kalayanarooj S., Green S., Vaughn D.W., Nisalak A., Ennis F.A., Rothman A.L. (2002). High circulating levels of the dengue virus nonstructural protein NS1 early in dengue illness correlate with the development of dengue hemorrhagic fever. J. Infect. Dis..

[137] Perera R., Khaliq M., Kuhn R.J. (2008). Closing the door on flaviviruses: Entry as a target for antiviral drug design. Antiviral Res..

[138] Zhou Z., Khaliq M., Suk J.E., Patkar C., Li L., Kuhn R.J., Post C.B. (2008). Antiviral compounds discovered by virtual screening of small-molecule libraries against dengue virus E protein. ACS Chem. Biol..

[139] Byrd C.M., Dai D., Grosenbach D.W., Berhanu A., Jones K.F., Cardwell K.B., Schneider C., Wineinger K.A., Page J.M., Harver C. (2013). A novel inhibitor of dengue virus replication that targets the capsid protein. Antimicrob. Agents Chemother..

[140] Sung C., Kumar G.S., Vasudevan S.G. (2014). Dengue Drug Development. Dengue and Dengue Hemorrhagic Fever.

[141] Chen Y.L., Abdul Ghafar N., Karuna R., Fu Y., Lim S.P., Schul W., Gu F., Herve M., Yokohama F., Wang G. (2014). Activation of peripheral blood mononuclear cells by dengue virus infection depotentiates balapiravir. J. Virol..

[142] Gubler D.J. (1998). The global pandemic of dengue/dengue haemorrhagic fever: Current status and prospects for the future. Ann. Acad. Med. Singap..

[143] Halstead S.B., Deen J. (2002). The future of dengue vaccines. Lancet.

[144] Utarini A., Indriani C., Ahmad R.A., Tantowijoyo W., Arguni E., Ansari M.R., Supriyati E., Wardana D.S., Meitika Y., Ernesia I. (2021). Efficacy of Wolbachia-Infected Mosquito Deployments for the Control of Dengue. N. Engl. J. Med..

[145] Bertsy G., Saleh M., Giner A., Lopez-Moya J.J., Lakatos L., Tanguy M., Pfeffer S., Haasnoot J., Berkhout B., Fuchs G. (2010). RNA Interference and Viruses: Current Innovations and Future Trends.

[146] Noble C.G., Shi P.Y. (2012). Structural biology of dengue virus enzymes: Towards rational design of therapeutics. Antiviral Res..

[147] Parameswaran P., Liu Y., Roskin K.M., Jackson K.K., Dixit V.P., Lee J.Y., Artiles K.L., Zompi S., Vargas M.J., Simen B.B. (2013). Convergent antibody signatures in human dengue. Cell Host Microbe.

[148] Liberis E., Velickovic P., Sormanni P., Vendruscolo M., Liò P. (2018). Parapred: Antibody paratope prediction using convolutional and recurrent neural networks. Bioinformatics.

[149] Deac A., VeliČković P., Sormanni P. (2019). Attentive Cross-Modal Paratope Prediction. J. Comput. Biol..

